# Evaluation of grain boundaries as percolation pathways in quartz-rich continental crust using Atomic Force Microscopy

**DOI:** 10.1038/s41598-021-89250-z

**Published:** 2021-05-10

**Authors:** Ritabrata Dobe, Anuja Das, Rabibrata Mukherjee, Saibal Gupta

**Affiliations:** 1 Department of Geology and Geophysics, IIT Kharagpur, Kharagpur, West Bengal 721 302 India; 2 Instability and Soft Patterning Laboratory, Department of Chemical Engineering, IIT Kharagpur, Kharagpur, West Bengal 721 302 India

**Keywords:** Geology, Structural geology, Solid Earth sciences, Petrology, Materials science, Microscopy, Atomic force microscopy

## Abstract

Hydrous fluids play a vital role in the chemical and rheological evolution of ductile, quartz-bearing continental crust, where fluid percolation pathways are controlled by grain boundary domains. In this study, widths of grain boundary domains in seven quartzite samples metamorphosed under varying crustal conditions were investigated using Atomic Force Microscopy (AFM) which allows comparatively easy, high magnification imaging and precise width measurements. It is observed that dynamic recrystallization at higher metamorphic grades is much more efficient at reducing grain boundary widths than at lower temperature conditions. The concept of force-distance spectroscopy, applied to geological samples for the first time, allows qualitative estimation of variations in the strength of grain boundary domains. The strength of grain boundary domains is inferred to be higher in the high grade quartzites, which is supported by Kernel Average Misorientation (KAM) studies using Electron Backscatter Diffraction (EBSD). The results of the study show that quartzites deformed and metamorphosed at higher grades have narrower channels without pores and an abundance of periodically arranged bridges oriented at right angles to the length of the boundary. We conclude that grain boundary domains in quartz-rich rocks are more resistant to fluid percolation in the granulite rather than the greenschist facies.

## Introduction

A number of mechanisms have been proposed in material and geological sciences to explain how fluids percolate through a medium under ductile and brittle conditions^1–10^. Most postulated mechanisms agree that in a 3-dimensional material framework, grain/phase boundaries play a major role in fluid percolation^7,11,12^. In the metallurgical and material sciences, it is well known that fluid percolation leads to corrosion or cracking; to circumvent these effects, attempts have been made to impart certain beneficial characteristics to the grain boundary network and make the material more resistant, a process referred to as ‘Grain Boundary Engineering (GBE)’^13,14^. In general, resistance to corrosion is controlled by the presence of ‘Coincident Site Lattice (CSL)’ boundaries between grains, which are basically high angle grain boundaries with additional periodic matches on lattice sites determined by a particular axis and angle of mismatch^15^ which impart certain ‘special’ properties to materials. In such cases, it has been suggested that small deformation followed by annealing (single step or iterative) may improve grain boundary properties, by increasing the proportion of CSL boundaries^16,17^.

In the geological sciences, fluid percolation has been investigated in mono-mineralic rocks like quartzites, as the mineral quartz constitutes much of the continental crust. Kruhl et al.^2^ estimated variations in the widths of grain boundary domains in quartzites metamorphosed at different metamorphic grades using a Transmitted Electron Microscope (TEM). While TEM is a very high resolution and high magnification technique, it is destructive in nature and involves an elaborate sample (wafer) preparation technique using a Focussed Ion Beam (FIB), which limits both its accessibility and convenience. In this study, we suggest Atomic Force Microscopy (AFM) as an alternative non-destructive method to study grain boundary morphology in geological materials. AFM enables nano-scale imaging of a sample surface directly from a thin section and gives additional information about the depth profile. While surface morphology imaging using AFM is routinely used in Materials Science and Nanotechnology^18,19^, it has only rarely been applied in geosciences, e.g. to study *in-situ* dissolution precipitation of minerals such as barite, celestite and calcite and for studying calcite surface structure^20,21^. It has also been used to study grooving and wetting along grain boundaries in quartz during hydrothermal annealing^4^. In this study, we demonstrate that systematic changes in grain boundary morphology in quartzite samples metamorphosed at different metamorphic grades can be directly visualized using AFM. We show that high resolution imagery of grain boundary domains using AFM permits determination of grain boundary features with precision, and suggest that this technique may be used for relating the width of grain boundaries to fluid percolation. Finally, we argue that these changes have implications for fluid flow in the middle and lower continental crust.

### Sample description

**Figure 1:**
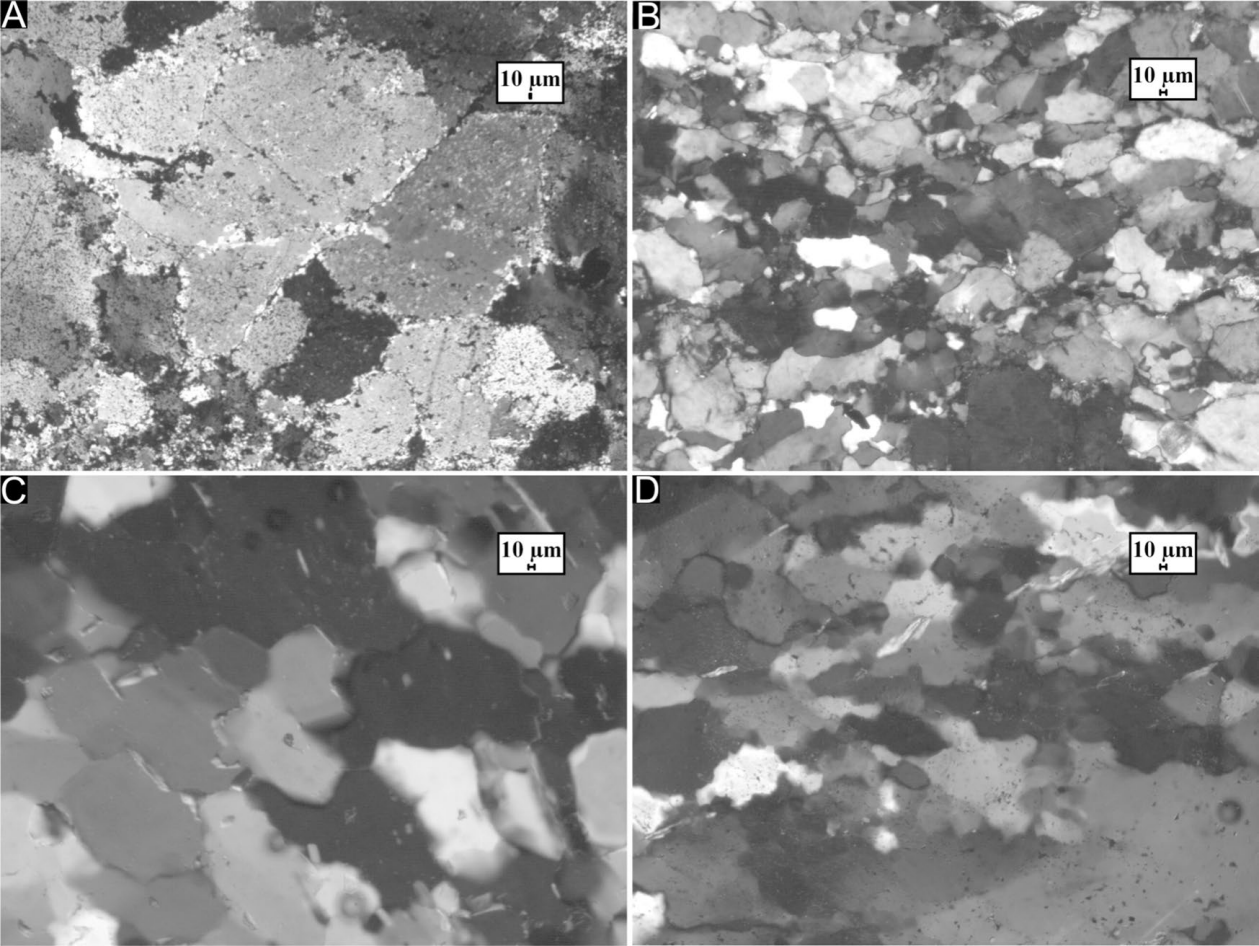
Thin section photomicrographs of four representative quartzite samples used for the purpose of this study, generated using Leica Qwin version 3 (Leica Microsystems); (**A**) RD 47: predominantly sedimentary quartzite from the Singhbhum Craton with limited deformation overprint. The average grain size is large but incipient recrystallization results in the development of prominent core-mantle structures with highly irregular grain boundaries. (**B**) RN 192: sheared quartzite from the Rengali Province. The average grain size is small and quartz grains are flattened and stretched in the direction of shearing, with prominent dextral asymmetry. (**C**) RN 36: quartzite from the Rengali Province which records a dominantly amphibolite facies deformation overprint. Grains are polygonal and equant with relatively straight boundaries implying deformation by dominantly static recrystallization. (**D**) RN 235: quartzite from the granulite unit of the central Rengali Province. Grains are amoeboid with highly irregular boundaries and comparatively larger sizes. Dynamic recrystallization at high temperatures implies that Grain Boundary Migration was the dominant deformation mechanism.

To eliminate variations originating from differences in mineralogy, seven relatively pure quartzite samples metamorphosed at different grades have been chosen for the purpose of this study. A polishing regimen similar to one used for Electron Backscatter Diffraction (EBSD) analyses has been used wherein the samples have been polished initially to a thickness of 30 microns on a polishing plate followed by polishing using colloidal silica for 6 h to remove surficial imperfections. The samples have been collected from the vicinity of a major terrane boundary zone in the eastern part of the Indian shield—specifically, between the Singhbhum Craton, the Rengali Province and the Eastern Ghats Province^22^ (see Supplementary Figure S1). The tectonothermal histories as well as the geochronological evolution of these terranes have been described in detail elsewhere^23–28^. Samples collected during fieldwork have been studied using a Leica DM-LP-4500 microscope. The metamorphic conditions experienced by the quartzites used in this study range from greenschist to granulite facies. RD 47 is an originally sedimentary quartzite metamorphosed under greenschist facies conditions, collected from the southern margin of the Singhbhum Craton. The quartz grains are polygonal in shape and evidence of strain retention is limited to Bulging Recrystallization (BLG)^29,30^ along with Grain Boundary Area Reduction (GBAR). Comparatively high strain zones within the quartzite are characterised by sub-grain rotation (SGR) and the development of core-mantle structures, with larger relict grains of quartz surrounded by smaller, recrystallized grains (Fig. 1A).

Quartzites of the Rengali Province are significantly different in character from those in the Singhbhum Craton. The intensity of deformation, as well as the metamorphic grade is higher than that experienced by the samples from the Singhbhum Craton^31^. High grade (amphibolite to granulite facies) metamorphism of Archean age accompanied pervasive fabric formation and annealing, which was followed by intense shearing along the terrane margins of the Rengali Province under greenschist facies conditions around ~ 500 Ma^24^. In samples from a sheared quartzite unit of the Rengali Province (RN 178, RN 192 and RN 201), quartz shows evidence of widespread dynamic recrystallization which accompanied dextral strike-slip shearing under greenschist facies conditions^24^. Mylonitization and accompanying grain size reduction is a characteristic feature of these sheared quartzites (Fig. 1B). Rotated kyanite porphyroclasts in RN 201 survive within the sheared matrix and serve as shear sense indicators. Away from the sheared domains, the effect of shear-related recrystallization gradually diminishes. Quartz grains in RN 36 from the Rengali Province are equant in shape, show undulose extinction^29^, and largely preserve the earlier, annealed granoblastic mosaic texture (Fig. 1C), although the marginal parts are modified by Bulging Recrystallization during later shearing. The texture is characteristic of static recrystallization followed by annealing at high temperatures. In the interior of the Rengali Province, the quartzites show evidence of high temperature recrystallization without any subsequent low temperature overprint. RN 235 contains amoeboid grains (Fig. 1D) indicative of Grain Boundary Migration (GBM)^30^. RN 235 also contains sillimanite needles oriented along the long axis of quartz ribbons. Quartz grains within ANG 1, a quartzite collected from the Eastern Ghats Mobile Belt south of the Rengali Province shows chess-board extinction patterns consisting of rectangular subgrains with relatively straight boundaries characteristic of deformation above 550 °C^29,32^.

## Results

### Atomic Force Microscopy

**Figure 2:**
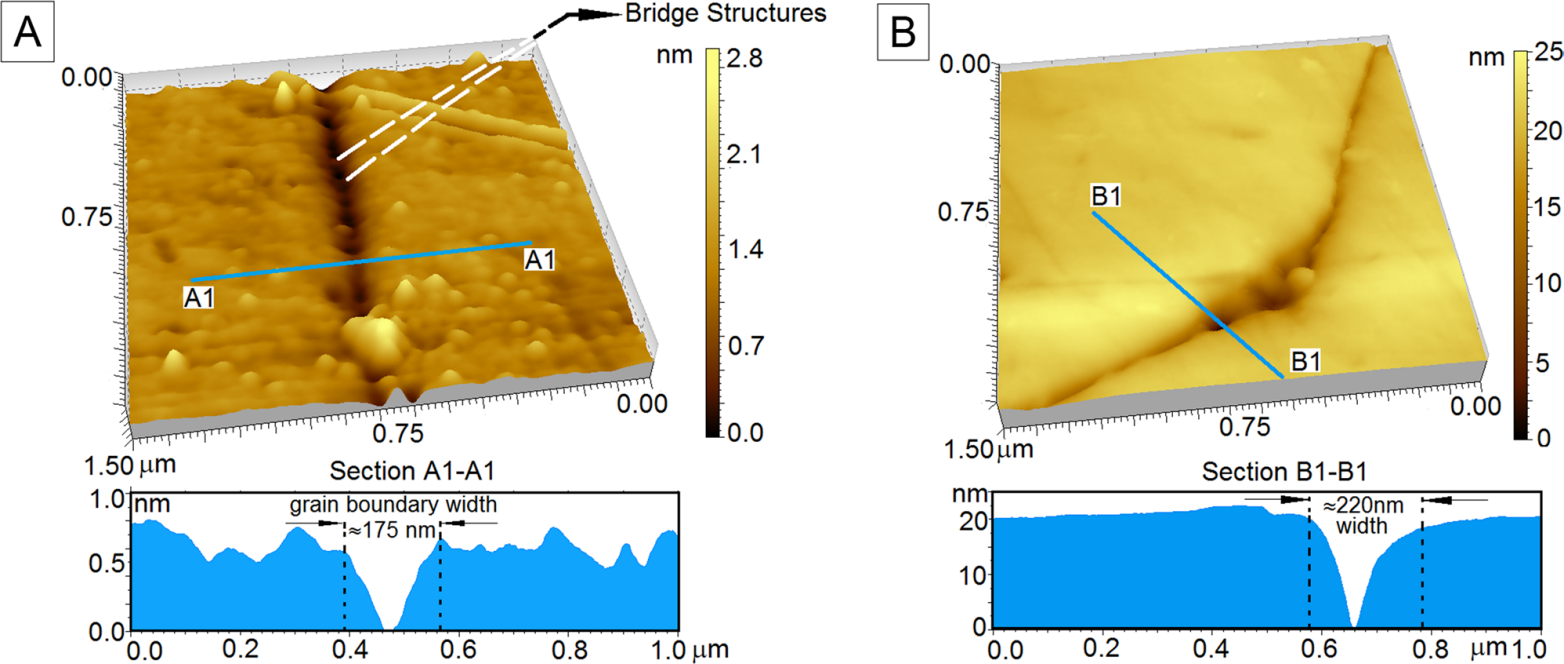
AFM scan images of two representative quartzite samples generated using PicoImage Basic Rendering Software (produced by Digital Surf), that comes integrated with Agilent PicoView Software (version 1.14.4, Agilent Technologies) URL:https://www.keysight.com/in/en/lib/software-detail/instrument-firmware-software/for-your-convenience-you-can-now-download-picoview-and-pico-image-software-for-your-afm-2282256.html. Depth profiles are shown below each scan image. Varying shades of yellow describe the surface elevation within each scan image; widths have been measured along the dotted lines in black which mark the inflection points. (**A**) Straight segment of a boundary within a quartzite deformed under granulite facies conditions (RN 235). Periodically arranged bridges, perpendicular to the length of the boundary are clearly observable. A polishing-induced scratch is visible in the top right corner of the image, which is slightly elevated with respect to the plane of the thin-section (**B**) Junction of two boundary segments within a quartzite deformed under amphibolite facies conditions (RN 36). The width of the junction is significantly larger than the individual boundary segments. Bridge structures are not apparent in this image. Very fine surficial imperfections are visible as linear features cutting across the pore at the junction of the boundary segments.

Width data estimated from a total of 441 grain boundary segments is presented in Table R1 (Section S2) of the Electronic Supplementary Information (ESI) with average widths mentioned in bold at the end of each column and calculated population standard deviation values mentioned in red. Grain boundary width has been computed as the distance along a straight line between successive inflection points on the AFM curve (see Fig. 2). Highest average grain boundary width (407.17 nm) is recorded in the statically recrystallized quartzite from the Rengali Province (RN 36), with a maximum width of 930 nm and a minimum width of 190 nm (Fig. 2B). The lowest average grain boundary width (97.25 nm) is recorded in a quartzite deformed and metamorphosed under granulite facies conditions (RN 235), with a maximum width of 212 nm and a minimum width of 34 nm (Fig. 2A). Widths show significant variation along the length of grain boundaries in greenschist facies samples, while they remain relatively consistent along boundaries in granulite facies samples. In the greenschist facies quartzite samples, the degree of dynamic recrystallization and the recrystallization regime in general (static vs dynamic) appears to influence grain boundary widths in the quartzites, with a greater degree of dynamic recrystallization accompanying smaller grain boundary widths, and vice versa. RD 47, which has limited imprint of dynamic recrystallization in the form of incipient sub-grain formation at the margins of large grains (see Fig. 1A), has an average boundary width of 323.19 nm, while the extensively sheared and recrystallized samples RN 178, RN 192 and RN 201 have average boundary widths of 364.34 nm, 339.05 nm, and 344.48 nm respectively.

The nature of grain boundary domains is not consistent within the scale of individual thin sections, and reveals a number of interesting features when imaged at high resolutions. Calculated standard deviation in grain boundaries from the greenschist facies samples RN 178, RN 192 and RN 201 are 378.72, 384.70, and 190.29 respectively. These values are significantly higher than the values calculated from the granulite facies samples ANG 1 and RN 235 (72.09 and 54.42). The large difference in standard deviation values between samples metamorphosed at different grades is due to the development of pore spaces at triple junctions and along individual segments of grain boundaries in low grade rocks. If these pores connect and create an inter-connected network, this could substantially ease percolation through these rocks. On the other hand, triple junctions in high grade rocks do not develop such pores but often consist of a comparatively wider boundary terminating at the junction of two comparatively narrower boundaries. While the average width is computed to be higher in the case of the statically recrystallized quartzite (RN 36), the widest grain boundary segments are present in the sheared (greenschist facies) quartzites which contain a number of pores along the grain boundary network, some of which are as wide as 1700 nm (see Table R1, Section S2, ESI).

High magnification imagery of the grain boundary domains reveals periodic ‘bridge’-type structures that are otherwise undetectable both in thin section and at lower magnifications under the AFM (Fig. 2A). These bridge structures are regularly arranged across the grain boundary voids and their abundance varies from sample to sample. 3D profiles of grain boundaries from two samples, RN 235 and RN 36 are shown in Fig. 2, where these bridges are clearly visible (Fig. 2A). The origin of these bridge structures may correspond to development of CSL boundary relationships described by McLaren^33^, especially because of their periodic distribution. To ensure that width measurements are not influenced by subjectivity in the choice of inflection points, the entire area occupied by representative boundary domains has been calculated. Calculated areas show no deviations from the trends observed in calculated widths across different metamorphic grades. The lowest average area of grain boundary domains is observed in RN 235 (0.086 µm^2^) while the highest average area is observed in RD 47 (19.974 µm^2^). ANG 1, the other quartzite sample which experienced granulite facies metamorphism, also contains smaller grain boundary domains on average (0.103 µm^2^) compared to RN 178 (3.155 µm^2^), RN 192 (1.666 µm^2^) and RN 201 (1.948 µm^2^) which were recrystallized under greenschist facies conditions. RN 36, which underwent static recrystallization, has an average grain boundary area of 0.956 µm^2^ which is intermediate between the values obtained for quartzites deformed under granulite and greenschist facies conditions. For the purpose of this study, boundaries between grains have been correctly identified and there are substantial morphological differences between grain boundaries as viewed under an AFM and surficial features such as polishing effects. Scratches due to polishing are typically limited in horizontal and vertical extent and individual scratches are straight and typically a few microns long and only a few nanometers deep (see Fig. 2, caption). Grain boundaries, on the other hand are arcuate, with locally short straight segments that extend vertically for several nanometers, showing only slight variations in width with depth. The boundaries exist entirely between grains and have been traced horizontally over several microns to ensure that they are not artefacts, such as healed fractures. Optical microscopy confirms the absence of any evidence of brittle deformation within the grains.

### Force-distance spectroscopy

**Figure 3:**
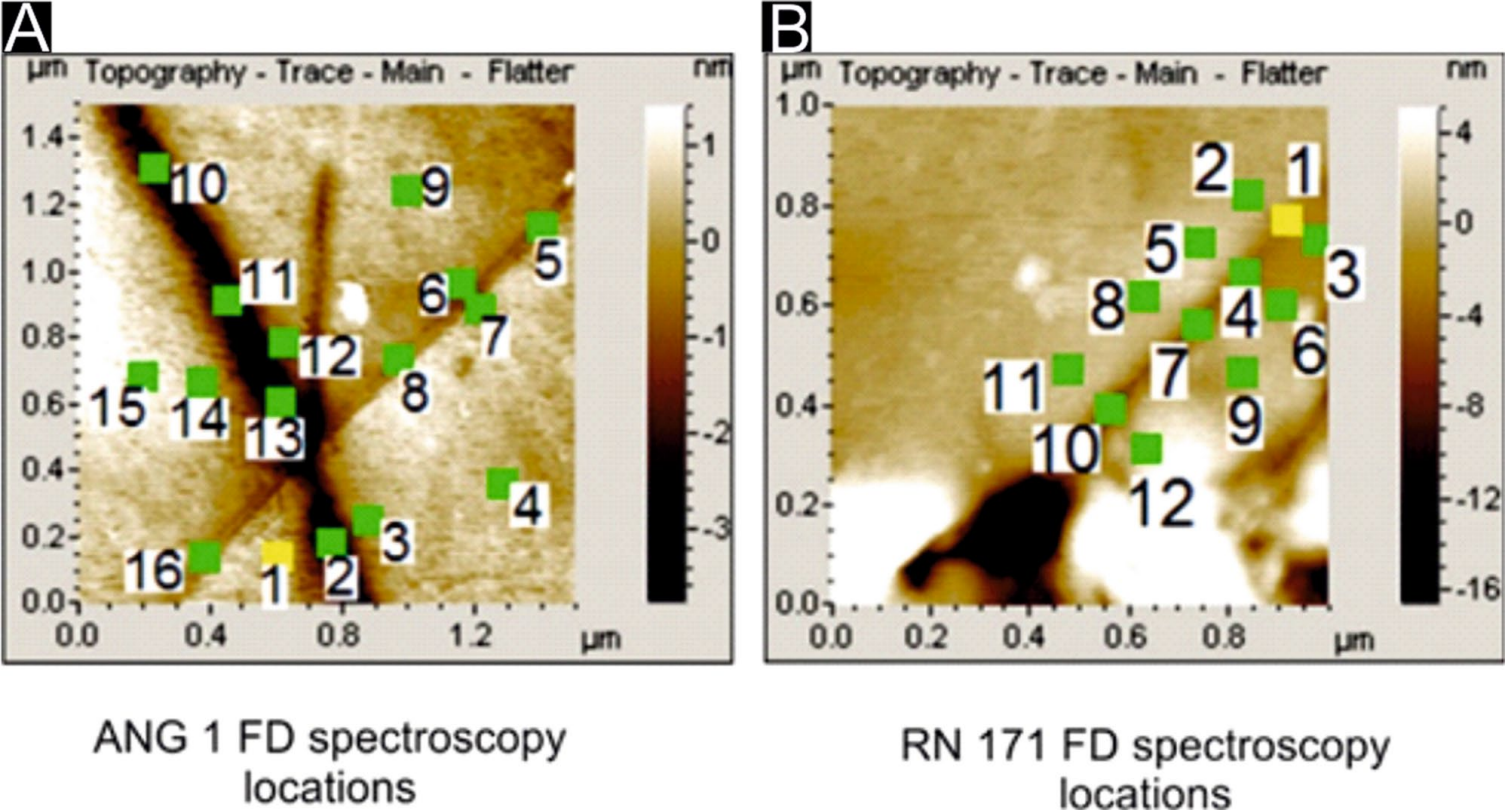
AFM images generated using PicoImage Basic Rendering Software (produced by Digital Surf), that comes integrated with Agilent PicoView Software (Version 1.14.4, Agilent Technologies) URL: https://www.keysight.com/in/en/lib/software-detail/instrument-firmware-software/for-your-convenience-you-can-now-download-picoview-and-pico-image-software-for-your-afm-2282256.html. Further, for better visual clarity, the numbers and the line profiles were drawn using MS Paint. Indent locations from which force-distance spectra have been obtained for two samples (A) ANG1 (B) RN171 are shown.

Table 1 presents representative calculated deformation data using force-distance (FD) spectroscopy for ANG 1 and RN 171. Corresponding AFM images marked with indent locations, generated using the FD module of the AFM software, are shown in Fig. 3. Additional deformation data obtained from FD spectroscopy at multiple locations for both samples have been listed in Table R2 and R3 (Section S3 of ESI). It is to be noted that all the FD curves were generated by the same AFM tip and on the same day to avoid any change in the material of the tip due to ambient conditions. The deformation of the sample surface is significantly greater in the case of RN 171 compared to ANG 1, and this has important implications for grain boundary character. A total of 36 points in ANG 1 and 21 points in RN 171 have been chosen for the purpose of force-distance spectroscopy (some of which are shown in Table 1, remaining in Section S3 of ESI), and these points are evenly distributed across the interior of the grains as well as along grain boundary domains. The overall deformation of the surface due to interaction with the tip, in case of ANG 1, is significantly less than RN 171, in addition to variations in deformation within a sample depending upon the location of the point being analysed. Within ANG 1, the difference in degree of deformation between points on the grain boundary (containing bridge structures) and the interior of the grain is small (e.g. 62.8 nm vs 66.3 nm), whereas the difference is significantly larger in the case of RN 171 along segments where bridges are absent. To emphasize the variation in intermolecular force over the bridge and non-bridge areas of one particular grain boundary, FD spectra have been obtained along grain boundary segments in RN 171, where bridges are present as well as those along which bridges are absent. Segments where bridges are present show smaller differences in extent of deformation between the interior of the grains and over the bridges, while segments where bridges are absent show much greater variability in degree of deformation between grain interiors and boundary domains. This implies that the deformation may be influenced by the presence of the bridge structures described earlier. The significantly higher population of bridge structures along grain boundaries in ANG 1 makes it more difficult for the tip to deform the surface significantly in comparison to grain boundaries in RN 171, in which the relatively scant occurrence of bridges makes it less rigid, thereby allowing the tip to deform the sample more. The deformation across all points for ANG 1 is one order less than that experienced by RN 171, from which it can be inferred that intermolecular strength at grain boundaries is stronger for ANG 1 than RN 171. The FD spectra curves have been used to calculate the average elastic moduli for ANG 1 and RN 171 (for details see section S5 of the ESI). ANG 1 has an average elastic modulus of 255 GPa within grains, and an average elastic modulus of 216 GPa in the grain boundary domains. The average elastic modulus within grains of RN 171 is 95 GPa and in the grain boundary domains, the average elastic modulus is 115 GPa. The elastic modulus is representative of the stiffness of a sample, or the resistance offered by a sample to deformation. The elastic modulus, both within grains and in grain boundary domains of ANG 1 is significantly higher than that in RN 171, implying that ANG 1 is more resistant to deformation than RN 171. This implies that granulite facies metamorphism results in significantly stronger bonds between constituent mineral grains compared to greenschist facies metamorphism.

**Table 1:** Results of plastic deformation calculated using force-distance spectroscopy on ANG 1 and RN 171. The deformation of the surface in case of ANG 1 is significantly lesser than that of RN 171 implying greater strength of the surface of ANG 1 in all domains, compared to RN 171.

Location	ANG 1 (nm)	RN 171 (nm)
1	66.3	175.02
2	62.8	43.8
3	54.6	180.9
4	57	630.9
5	60.7	123.7
6	54.9	376.5
7	65.5	738.7
8	61.1	619.1
9	75.8	752.2
10	56.9	319.7
11	67.4	564.9
12	51	493
13	60.2	
14	60.6	
15	61.1	
16	62

### EBSD studies

**Figure 4:**
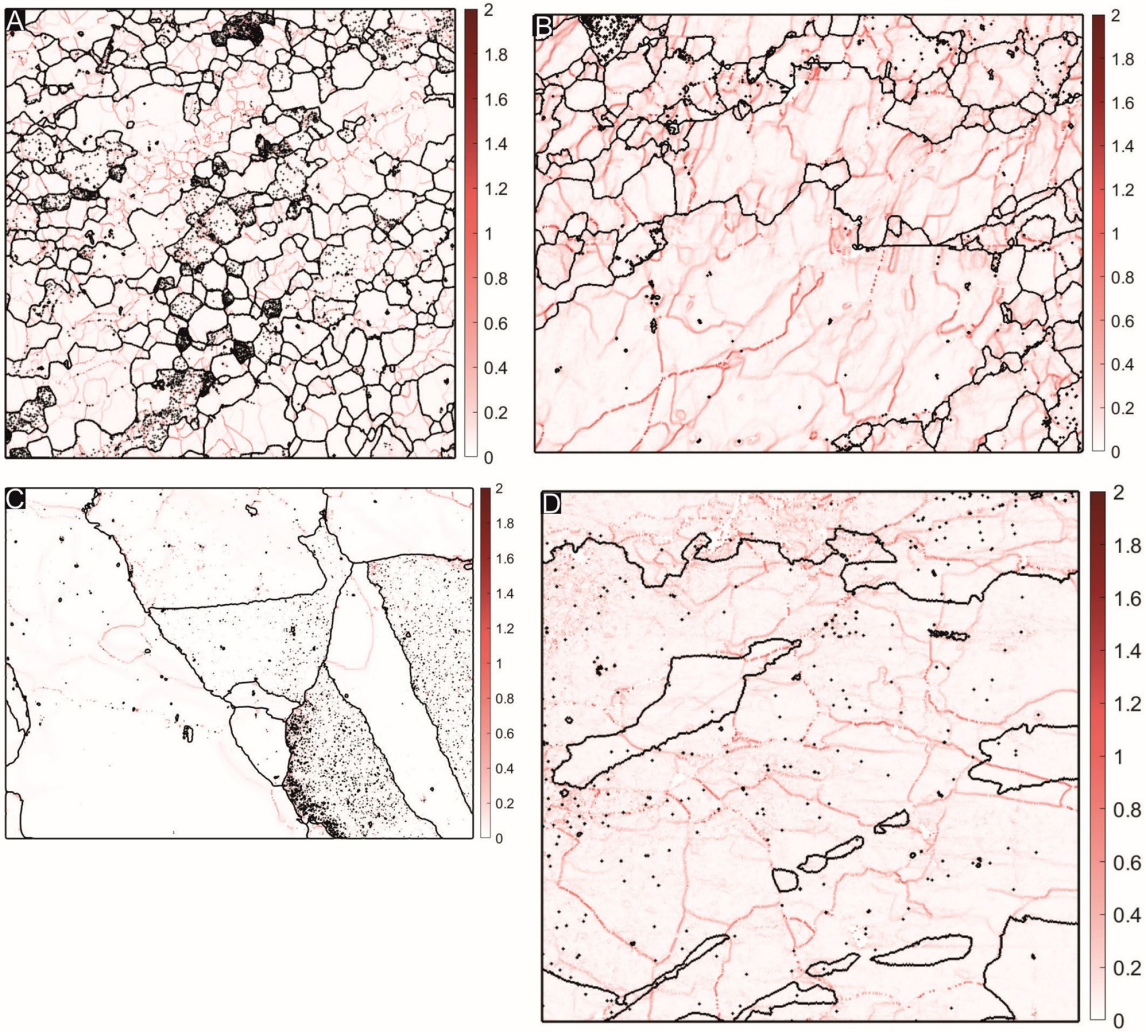
Kernel Average Misorientation maps of four representative quartzite samples used in this study, generated using the software package Mtex version 5.6.0 for Matlab. (Matlab version 2020a). URL: https://mtex-toolbox.github.io/. Varying shades of red describe the misorientation angle at different points in the map; (**A**) RN 178: sheared quartzite from the Rengali Province. Larger grains have a significantly higher amount of strain (dislocations) compared to smaller grains, as is expected for incomplete dynamic recrystallization. In some grains, dislocations are perpendicular to the grain boundary lengths. (**B**) RN 221: sheared quartzite from the Rengali Province. Comparatively larger grain size compared to A, because of which the density of dislocations is higher. Smaller grains are almost dislocation free whereas large grains have dense concentrations of dislocations within them. (**C**) RN 36: statically recrystallized quartzite from the Rengali Province. Almost strain free, with very few isolated dislocations in the grain interiors. Static recrystallization and accompanying polygonization without further strain imprint appears to have left this quartzite relatively strain free. (**D**) RN 235: quartzite deformed under granulite facies conditions. Large amoeboid grains with a significant dislocation density are the dominant feature of this quartzite. Smaller recrystallized grains appear to be relatively strain free.

Kernel Average Misorientations (KAM) are a useful tool for visualizing remnant strain in terms of dislocation density (see Fig. 4). KAM values have been computed using Mtex for MATLAB^34^ from (EBSD) data generated from four quartzite samples (RN 36, RN 178, RN 221 and RN 235), to understand the effect (if any) played by dislocation density on widening of grain boundary domains. A higher dislocation density would imply greater remnant strain and vice versa. The sheared quartzites (RN 178, Fig. 4A; RN 221, Fig. 4B) preserve a high degree of remnant strain in their microstructure, but most of it is restricted to intra-granular domains, away from grain boundaries. The statically recrystallized quartzite sample (RN 36, Fig. 4C) has the least remnant strain, since it was initially metamorphosed at higher, amphibolite facies conditions, and was in a low strain zone of the later greenschist facies shearing; only marginal effects along the grain boundary were impressed during this later event^24^. The quartzite recrystallized at high temperature (RN 235, Fig. 4D) has a significant amount of remnant strain in its microstructure, evidenced by dislocation build up within grains as well as a significant number of dislocation walls cross-cutting grain boundary domains. The results of the KAM analyses indicate that the proportion of remnant strain does not necessarily accentuate grain boundary widths during polishing and sample preparation. The sheared quartzites have wider grain boundaries, and have strain concentrated in the grain interiors away from boundary domains; in contrast, the high temperature quartzite has narrower grain boundaries but a significant dislocation density near grain boundary domains.

## Discussion

It has been suggested that open grain boundaries, cavities, and depressions can form a network which allows fluid circulation in rocks^2,7,11,35–37^. However, such cavities and pores in rocks are not uniformly distributed^8,11,38^. During metamorphism, it is known that textural equilibration significantly modifies grain boundary geometry^29^; however, there has been no systematic investigation as yet to determine if there is any correlation between the grain boundary width and the grade of metamorphism, which is one of the primary controls on rock texture, along with other factors such as the orientation of the grain boundary relative to local kinematics, stretching direction, or cavitation along specific boundaries, to name a few. Textural equilibration is accompanied by attainment of equilibrium dihedral angles at triple junctions of grains^38,39^ with a concomitant change in crystallographic orientations across grain boundaries; these changes are commonly determined by Electron Backscatter Diffraction (EBSD). During metamorphism, crystallographic re-orientation may in some cases lead to the formation of ‘special’ boundaries between grains, known as Coincident Site Lattice or CSL boundaries^15,40^. The mechanical process of migration of fluids along a grain boundary is controlled by the width of the channel, with a greater width facilitating easier fluid transport along the channel. If some of these boundaries are CSL boundaries, they offer enhanced resistance to fluid percolation along the grain boundary network^17,33,41,42^. Thus, in metallurgical and material science studies, the width of the grain boundaries, in terms of wider general boundaries and narrower CSL boundaries, are taken into consideration to model percolation behaviour in metals and materials^41,43^.

In this study, we estimated the width of the grain boundary domain using Atomic Force Microscopy (AFM), which also reveals other interesting morphological features along the boundaries. Comparison of grain boundaries in quartzites metamorphosed under conditions varying from the greenschist to granulite facies shows distinct differences. Quartzites metamorphosed under greenschist facies conditions have wider grain boundaries compared to those metamorphosed under granulite facies conditions. For all the dynamically recrystallized samples, the reduction of grain boundary width is correlatable with increasing metamorphic grade. This implies that lower grade rocks generally have wider grain boundary domains, with abundant pores along the grain boundary network, than high grade rocks, and are therefore more amenable to fluid flow. The wide grain boundary domains in one apparently discrepant sample in this study (RN 36) can be attributed to the effect of modification of statically recrystallized grain boundary domains during the later lower temperature shearing event in the Rengali Province. Apart from the grain boundary width, an important morphological feature observed across the grain boundaries are the bridge-type structures detected with the AFM under high magnification, suggesting enhanced bonding across these boundaries. These bridge-structures are exclusively observed along narrower grain boundary segments, and have not been previously documented with any alternative technique. The results of force-distance spectroscopy provide more insight into the significance of these bridges. On grain boundary segments that contain bridges, the bridge domains show significantly less deformation on interaction with the AFM tip compared to the domains between the bridges. This suggests that there is a spatial variation in bond strength along these grain boundaries, being significantly higher across the bridges than in inter-bridge segments. Additionally, force-distance spectroscopy on one low grade and one high grade sample also suggests that bonding across grain boundaries in high grade rocks are significantly stronger than in low grade rocks. The enhanced bonding strength, and the presence of bridges across grain boundaries in high grade rocks increase the bulk strength of the rock and improve resistance to fluid percolation; they may also be regarded as evidence for the existence of special (CSL) boundaries in rocks, similar to those reported in metals. The most common such CSL relationship observed in quartz rich rocks are Dauphiné twins which involve a 60° rotation about the <0001> axis^33,44–48^.

Since the present study attempts to relate grain boundary domains in metamorphic rocks of different grades to fluid flow, it is pertinent to discuss if the grain boundary widths measured under ambient pressure temperature conditions are representative of the in situ situation at depth. Studies on quartz^49–52^ show that volume reduction associated with decompression is anisotropic. This implies that quartz grains would undergo volume reduction as they cool through the brittle-ductile transition during uplift, with associated widening of grain boundaries. However, the results of this study argue that for high grade and low grade rocks, the situation should be different. The enhanced degree of bonding (manifested as bridges under an AFM) in high grade rocks would imply that the constriction of grains on either side of the boundary is limited, and does not necessarily influence the boundary width itself. Additionally, strong bonds across a grain boundary would prevent the boundary from widening or forming voids due to decompression, to a much larger extent than boundaries across which the bonds are weak. The width of boundaries that contain bridge structures are therefore inferred to preserve their original widths from depth to the surface, and are not an artefact of exhumation. Thus, it appears that in quartzites, grain boundary widths reduce significantly with increasing metamorphic grade, with enhanced bonding at higher grades forming ‘bridge-type’ structures across the grain boundaries. Ultimately, this implies an increased resistance of channels in quartz-rich high grade rocks to fluid flow, consistent with the relatively anhydrous character of the granulite facies. Enhanced bonding across most grain boundaries in high grade rocks reduces the degree of opening of grain boundaries on cooling and decompression, whereas the more limited amount of bonding in lower grade rocks means these boundaries open more; therefore, difference in observed widths reflect the difference in bonding and its influence on grain boundary opening.

## Conclusions

Atomic Force Microscopy allows comparatively easy, high magnification imaging and precise width measurements along grain boundary domains within a rock. Within quartzites deformed and metamorphosed at different metamorphic conditions, those that are deformed at lower grades have wider grain boundaries with voids along the channels and at triple junctions and a much lower proportion of bridges compared to those that are deformed at higher grades. The latter have narrower channels, no voids along these channels, and an abundance of periodically arranged bridges oriented at right angles to the length of the boundary. The width of a grain boundary is not constant laterally and shows significant variability along the length of the boundary. The effect of the dominant recrystallization mechanism (dynamic vs static) on grain boundary widths has not been studied previously, and this study suggests that dynamic recrystallization results in much narrower grain boundaries compared to static recrystallization. Thus, dynamic recrystallization at higher metamorphic grades is much more efficient at reducing grain boundary widths compared to static recrystallization under similar or slightly lower temperature–pressure conditions. The concept of force-distance (FD) spectroscopy has been applied to geological samples for the first time, and the results allow for a qualitative estimate of variations in strength of grain boundary domains and grain interiors within quartzites deformed at different metamorphic conditions. The results of FD spectroscopy demonstrate that high grade rocks are in general significantly less deformable compared to low grade rocks. Additionally, grain boundary domains containing bridges across them are less deformable compared to segments along which bridges are absent, irrespective of metamorphic grade. We suggest that the strength of grain boundary domains is higher in high grade metamorphic rocks compared to lower metamorphic grade rocks, and together with the other grain boundary features, contribute to enhanced resistance to fluid percolation in granulite facies quartzites compared to those metamorphosed under greenschist facies conditions.

## Methods

### Atomic Force Microscopy

Surface morphologies of the seven quartzite samples were studied using an Atomic Force Microscope (*Model 5100, Agilent Technologies*). An Atomic Force Microscope images the surface of a ‘material’ based on minute variations in elevations of the sample surface relative to the deepest point that the AFM tip can probe for a specific sample. A *n doped*—Silicon Nitride (*PPP-NCL, Nanosensors Inc., USA*) four sided AFM tip attached to a silicon cantilever, having a tip radius of curvature of 8 ± 2 nm, cantilever resonating frequency in the range of 180–250 kHz and force constant of 48 N/m, records the variation in topography at the surface. All AFM scans are obtained in intermittent contact mode (widely known as tapping mode) to ensure there is no damage made to either the AFM tip or the surface of the sample. Scans have been generated by Raster Scanning at a speed of 0.3 lines per second. Since pure quartzites (containing more than 90% quartz) have been used in this study, all grain boundary width data relate to boundaries between adjacent quartz grains. Additionally, the quartzites have been studied under an optical microscope to delineate domains free of accessory minerals, and these domains have been further imaged using the AFM. The darkest zones are the deep trenches in the image and are obviously grain boundaries (see Fig. 2); depth profiles were obtained across them using the AFM. Grain boundary widths are then computed from these depth profiles.

### Force Distance Spectroscopy

Force-distance spectra have been obtained using the AFM both over grain boundary domains, and in the interior of grains. A total of 36 points for ANG 1 and 21 points for RN 171 have been identified by first imaging the area and then capturing the FD spectra at each point. Details with regard to this technique have been outlined in Cappella and Dietler^53^. Out of several extractable parameters, which describe the sample surface with the help of Force—Distance (FD) Spectroscopy, a property known as ‘Plastic Deformation’ has been calculated in this study, which can qualitatively explain variations in intermolecular forces within the grain boundary domains, as well as variations in intermolecular forces between grain boundary domains and the grain interior. This is actually deformation that occurs and recovers gradually over time depending upon the elasticity of the material, and indicates that there is ongoing deformation at that particular point within the observation time-scale or time window during which the FD curves are being generated. Lesser deformation implies stronger grain boundary structures. In addition to this, elastic modulii (***E***_***S***_) of locations within the grains and near the grain boundaries have been calculated for both samples; detailed calculation procedures are shown in Section S5 of the ESI. To obtain the FD spectra, the applied force on the AFM tip has been kept constant throughout the study at ≈ 6.84 µN (equivalent to 9.5 V on photodiode) (calculation of the applied force from electrical signal generated on photodiode is shown in Section S4 of ESI).

### EBSD studies

Electron Backscatter Diffraction (EBSD) data have been generated using a Zeiss-Auriga Compact system with a Gemini column Schottky type field emission filament. EBSD pattern detection is carried out using an Oxford Nordlys detector. The EBSD analyses have been carried out using a voltage of 30 kV and a step size of 0.5 microns. Grains within the EBSD map have been delineated using a threshold angle of 10° and from indexed grains only. Raw EBSD data have been de-noised using a half-quadratic filter and the KAM threshold has been set as 2.5° to document sensitive local misorientations, which provide an estimate of the dislocation density in a region.

## Supplementary Information

Supplementary Information
