# Supplementary material for: Evaluation of grain boundaries as percolation pathways in quartz-rich continental crust using Atomic Force Microscopy
Source: Sci Rep. 2021 May 10;11:9831. doi: 10.1038/s41598-021-89250-z (PMC8111022; doi:10.1038/s41598-021-89250-z)
Supplement: Supplementary file 1 — Supplementary Information [file 41598_2021_89250_MOESM1_ESM.pdf]

Evaluation of grain boundaries as percolation pathways in quartz-rich continental crust using Atomic Force Microscopy

Ritabrata Dobe[1], Anuja Das[2], Rabibrata Mukherjee[2] and Saibal Gupta[1]*

(Email: saibl2008@gmail.com)

[1]Department of Geology & Geophysics, IIT Kharagpur, Kharagpur, West Bengal, India – 721 302

[2] Instability and Soft Patterning Laboratory, Department of Chemical Engineering, IIT Kharagpur, Kharagpur, West Bengal, India – 721 302

S1: SUPPLEMENTARY FIGURE S1

Schematic regional map showing the locations of and contacts between the Singhbhum Craton, Rengali Province and Eastern Ghats Mobile Belt drawn using Corel Draw X5.

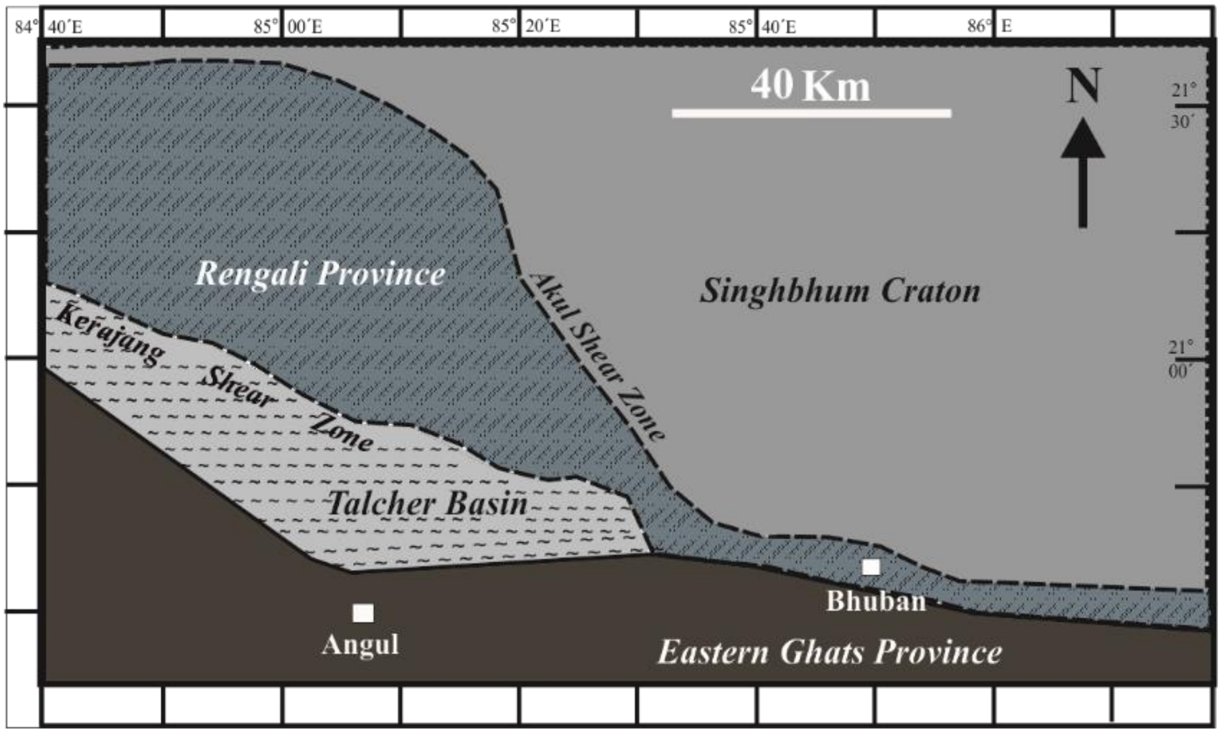

S2. Width of Grain Boundaries for each sample

Table R1: Grain Boundary Width Measurements (Average width values mentioned in **bold**, Standard deviation values mentioned in red)

ANG1 (nm)	RD47 (nm)	RN36 (nm)	RN178 (nm)	RN192 (nm)	RN 201 (nm)	RN 235 (nm)
275	387	270	215	200	215	199
280	354	350	220	155	255	190
210	355	390	200	95	240	194
215	333	309	205	185	225	207
225	333	305	230	207	322	212
270	310	285	245	210	325	195
290	317	265	182	173	350	160
225	305	305	207	165	425	165
215	328	285	228	155	490	185
280	355	595	170	185	370	186
250	377	560	202	183	333	181
205	358	810	332	177	536	185
215	373	790	280	148	248	150
220	380	710	345	145	442	160
200	320	520	630	140	595	153
207	300	575	1010	143	600	170
200	264	273	925	140	595	157
125	280	292	1275	150	606	157
130	262	290	1475	130	620	140
135	288	550	1458	167	555	178
138	250	680	1300	172	655	137
145	285	575	1462	187	540	75
55	335	190	1350	162	635	63
50	280	280	1030	170	720	68
64	315	288	1010	183	365	53
72	283	255	602	210	740	63
102	280	415	215	236	640	68
95	310	270	217	222	570	62
112	365	305	190	170	215	65
110	318	260	250	122	210	67
110	330	230	137	158	124	89
123	288	375	160	112	128	96
110	315	390	165	110	150	92
106	342	255	152	395	102	78
100	285	290	68	360	150	70
88	255	305	167	345	155	78

102	275	338	137	280	235	101
112	323	320	190	300	250	96
110	310	290	152	323	242	78
73	265	270	155	220	262	82
92	327	330	113	750	240	83
123	302	343	147	750	232	62
104	285	350	145	950	110	72
79	256	540	158	650	225	73
84	230	858	175	1155	105	84
250	275	930	112	1260	207	84
265	247	540	110	1550	152	69
240	272	390	140	1700	197	83
250	242	360	100	1500	103	55
230	243	490	120	1000	218	62
**161.32**	225	400	170	114	**344.48**	73
72.0875689 7	195	320	155	128	190.286 1	57
	192	315	142	170		63
	170	450	77	117		42
	235	245	115	138		38
	330	500	150	140		44
	305	470	132	108		47
	350	440	140	150		52
	285	430	205	154		39
	272	419	232	169		34
	363	**407.166666 7**	225	**339.05**		49
	293	165.371013 9	105	384.699771 5		42
	300		270			48
	340		190			46
	330		220			46
	420		310			47
	388		398			42
	345		520			35
	348		604			34
	307		615			**97.2463 8**
	270		430			54.4150 5
	217		**364.338028 2**			
	310		378.719969 5			
	412					
	447					
	450					

	577					
	603					
	513					
	540					
	580					
	**323.1975309**					
	81.55062349					

S3. Determination of Plastic Deformation from FD curves:

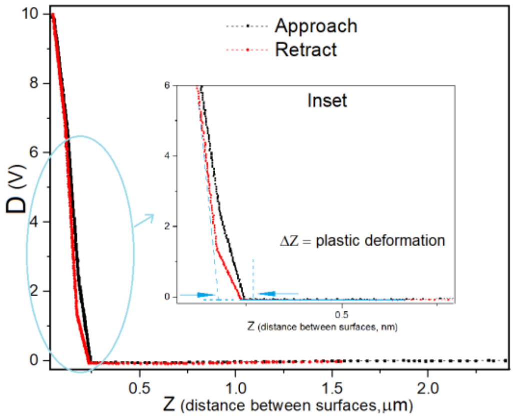

Figure S2. FD curve for location 5 (area 2) of ANG 1, showing determination of plastic deformation. Inset figure shows the magnified version of the encircled region of the FD curve.

Zero-load plastic deformation (ΔZ) is determined by the difference in the value of Z of approach curve and the tangent to the retraction curve which meets at zero deflection (or zero force, y = 0), shown in Figure S2 for sample ANG 1 (location 5, area 2), as previously reported by Cappella et.al.[1] Zero load plastic deformation data obtained at all locations for both samples (ANG 1 and RN 171) are listed below in table R2 and R3.

Table R2: Plastic Deformation Data for sample ANG 1 calculated at different locations for three different areas. The **i**mages have been generated using PicoImage Basic Rendering Software (produced by Digital Surf), that comes integrated with Agilent PicoView Software (Version 1.14.4, Agilent Technologies) URL: https://www.keysight.com/in/en/lib/software-detail/instrument-firmware-software/for-your-convenience-you-can-now-download-picoview-and-pico-image-software-for-your-afm-2282256.html. Further, for better visual clarity, the numbers and the line profiles were drawn using MS Paint.

Location	Area 1 (nm)	Area 2 (nm)	Area 3 (nm)
	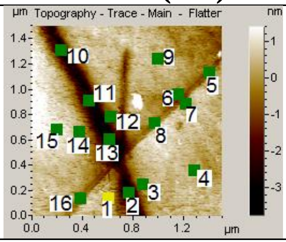	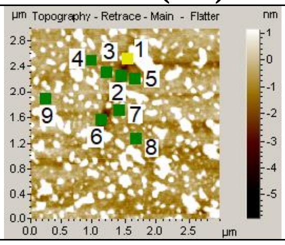	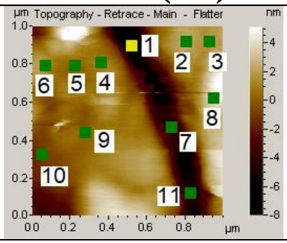
1	66.3	65.2	68.9
2	62.8	60.2	61.2
3	54.6	76.3	61.5
4	57.0	96.2	55.6
5	60.7	128.0	57.9
6	54.9	54.1	52.0
7	65.5	65.8	65.3
8	61.1	74.2	62.3
9	75.8	64.3	59.3
10	56.9		57.8
11	67.4		70.3
12	51.0		
13	60.2		
14	60.6		
15	61.1		
16	62.0		

Table R3: Plastic Deformation Data for sample RN 171 calculated at different locations for two different areas. The **i**mages have been generated using PicoImage Basic Rendering Software (produced by Digital Surf), that comes integrated with Agilent PicoView Software (Version 1.14.4, Agilent Technologies) URL: https://www.keysight.com/in/en/lib/software-detail/instrument-firmware-software/for-your-convenience-you-can-now-download-picoview-and-pico-image-software-for-your-afm-2282256.html. Further, for better visual clarity, the numbers and the line profiles were drawn using MS Paint.

Location	Area 1 (nm)	Area 2 (nm)
	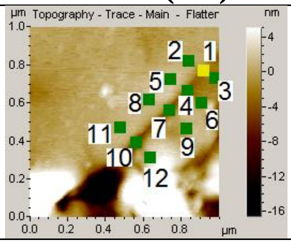	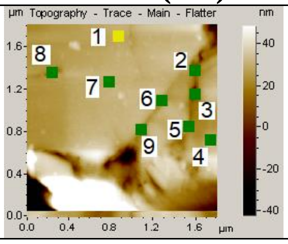
1	175.02	183.5

2	43.8	566.3
3	180.9	845.0
4	630.9	430.2
5	123.7	356.5
6	376.5	132.5
7	738.7	95.6
8	619.1	169.3
9	752.2	825.2
10	320.1	
11	564.9	
12	493.0	

S4. Calculation of applied force (P) using Hooke's Law:

To determine the applied force (***P***), the following tip properties are required:

Deflection sensitivity, **A** (as extracted from PicoView Spectroscopy software using rigid known sample) = 15 nm/V

Tip Force Constant, **k** (as provided by the manufacturer of AFM tips) = 48 N/m

Now, vertical tip deflection, ***δ`*** can be related to electrical signal (generated in photodiode in volts): [2]

$\delta` = A\,(D - D_0)$**……….(1)**

D is considered as the maximum deflection signal on FD curve (See figure S3) and $\mathbf{D_0}$ is the initial signal. From the FD curve, we obtain **D** = 10.0 V and $\mathbf{D_0}$ = 0.5 V and using **A** = 15 nm/V in equation 1, we obtain ***δ`* = 142.5 nm**.

Further, ***δ`*** can be utilized to calculate the applied force, ***P*** on the tip, which is typically modelled as a spring obeying Hooke's Law.[1, 2] Thus, force on the AFM tip can be related as, $P = k.\ \delta`$ **…. (2)** (where **k** = tip force constant in N/m). Using the values of **k** and ***δ`*** as mentioned above in equation 2, we get ***P*** = 6840 nN or 6.84 µN.

S5. Determination of Elastic Modulus:

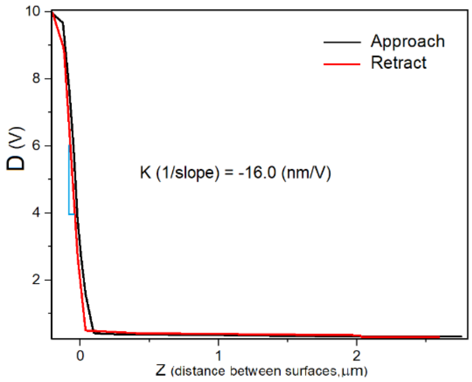

Figure S3. Representative distance vs deflection curve generated for rigid Silicon substrate (known sample).

Determination of elastic modulus of an unknown sample requires certain parameters that are inherent to the material and make of the tip. Deflection sensitivity (**A**) and cantilever-tip property ($\boldsymbol{\alpha}$) are determined from the PicoView software using a rigid sample (Silicon Wafer Substrate) as known sample and by calculating from the following equations:[2, 3]

$$\frac{1}{E_r} = \frac{(1-\vartheta_i)^2}{E_i} + \frac{(1-\vartheta_s)^2}{E_s} \text{......(3)}$$

$$|K| = A\left(1 + \frac{k}{2.E_r.a}\right) \text{...........(4)}$$

$$\alpha = \frac{k}{2.a} \text{.........................(5)}$$

$$E_r = \frac{\alpha}{\frac{|K|}{A} - 1} \text{..........................(6)}$$

Where: E_s, E_i and E_r = elastic modulus of sample, indenter (AFM tip) and reduced elastic modulus respectively, in Pascal. ϑ_i, ϑ_S = Poisson's Ratio of Indenter and Sample respectively. **K** = 1 / slope of retraction curve (nm/V), **A** = deflection sensitivity (nm/V), **k** = tip force constant (N/m), **a**= contact radius (nm), α = cantilever tip property in Pa.

Taking the values of E_s = 112.40 GPa and ϑ_S = 0.28 for known sample (Si Wafer substrate) from published literature[4,5] and the values of E_i = 310 GPa and ϑ_i = 0.27 for silicon nitride AFM tip,[6] we find E_r = 0.1579 x 10^{-12} Pa. This value of E_r is used to find $\boldsymbol{a}$ from equation 4, where |**K**| = 16 nm/V for Si substrate (determined from the FD retraction curve for Si substrate, See Figure S3). With the calculated value of $\boldsymbol{a}$ which is 2278.78 x 10^{-12} nm, we then find $\boldsymbol{\alpha}$ from equation 5, which is calculated to be ≈ 10.5 GPa.

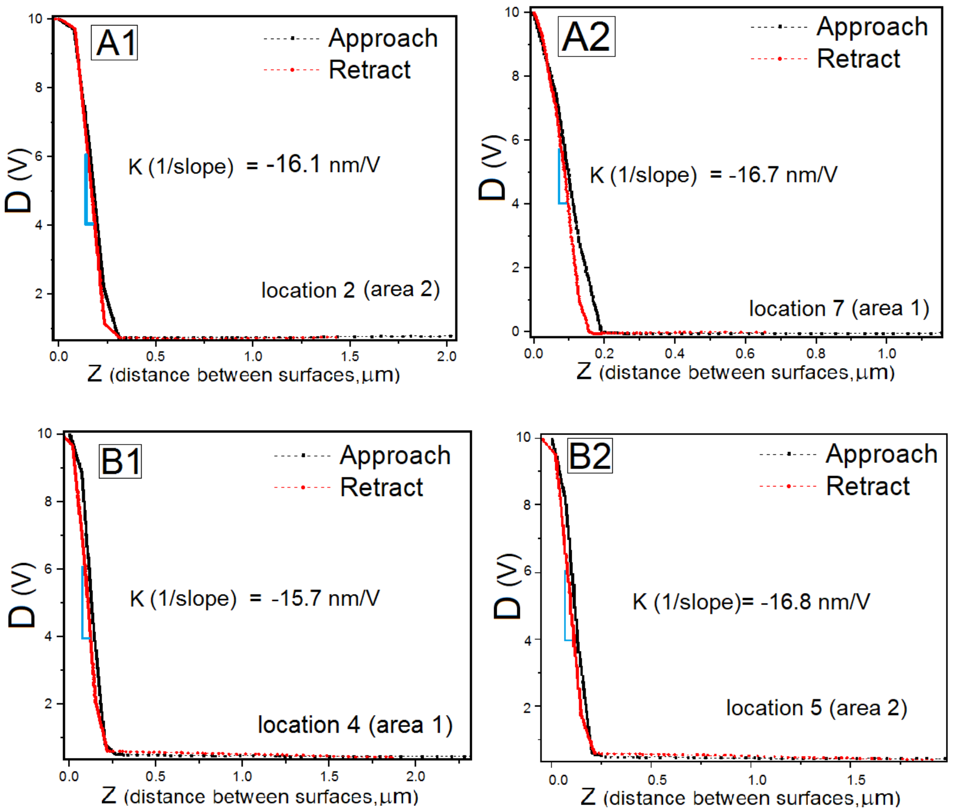

Figure S4. Representative distance vs deflection curve generated for (**Series A**) ANG 1; (**Series B**) RN 171

Further to determine ***Es*** of the samples (ANG 1 and RN 171), we have obtained $\boldsymbol{E_r}$ (from eq. 6) using the values of $\boldsymbol{\alpha}$ = 10.5 GPa and **A** = 15 nm/V and ***K***. The values of ***K*** have been determined from the average slope of the retraction curves generated for locations within grain boundaries and near the grain boundary of each sample (See figure S4). The value of $\boldsymbol{E_r}$, obtained, was used to calculate ***Es*** from equation 3, using the known values of $\boldsymbol{E_i}, \boldsymbol{\vartheta_i}$, as mentioned above and $\boldsymbol{\vartheta_S} \approx 0.107$ from literature[7].

Table R4: K-values (nm/V) for each location of sample ANG 1:

Location	Area 1	Area 2	Area 3
1	-14.9	-14.9	-14.6
2	-16.5	-16.1	-15.2
3	-15.7	-15.2	-14.9
4	-15.2	-14.9	-14.9
5	-14.9	-14.6	-15.6
6	-14.6	-16.7	-15.6
7	-16.7	-16.7	-15.2
8	-16.1	-14.8	-16.7
9	-17.1	-17.1	-14.6

10	-15.6		-17.1
11	-15.8		-14.9
12	-14.9		
13	-15.2		
14	-14.9		
15	-17.3		

As an example, below we show the calculation for obtaining elastic modulus (E_s) of location within the grain boundary of ANG 1:

From table R4, $|K|_{avg}$ value for location within the grain boundaries (considering all three areas of the sample, for example: location 2, 10, 13 of area 1) is calculated to be ≈ **15.75 nm/V** and $|K|_{avg}$ for locations surrounding the grain boundary (example: locations 1, 4, 7, 10 of area 1) is found to be ≈ **15.85 nm/V**

Using **α** = 10.5 GPa (as calculated above), $|K|_{avg}$ = 15.75 nm/V in equation 6

$$E_r = \frac{10.5}{\frac{15.77}{15} - 1}$$

⇨ E_r **= 204.54 GPa** (within grain)

For location surrounding grain boundaries $|K|_{avg}$ = 15.85 nm/V (See Table R4)

$$E_r = \frac{10.5}{\frac{15.85}{15} - 1}$$

⇨ E_r **= 185.29 GPa** (near grain boundaries)

Using E_r = **204.54 GPa** and **185.29** in equation 3 we get,

$$\frac{1}{204.54} = \frac{(1-0.27)^2}{310} + \frac{(1-0.107)^2}{E_s}$$

⇨ E_s **= 252.2 GPa** (within grain)

and,

$$\frac{1}{185.29} = \frac{(1-0.27)^2}{310} + \frac{(1-0.107)^2}{E_s}$$

⇨ E_s **= 216 GPa** (near grain boundaries)

Table R5. K-values (nm/V) obtained from retraction curves for sample RN 171

Location	Area 1	Area 2

1	-17.8	-16.2
2	-15.7	-16.2
3	-16.8	-17.1
4	-15.7	-15.7
5	-15.7	-16.8
6	-15.7	-15.7
7	-16.7	-17.3
8	-17.1	-17.1
9	-17.1	-17.3
10	-16.2	
11	-17.1	
12	-16.2	

For RN 171, using the same method for calculation of elastic modulus (E_s), as shown above, we get E_s (within grain) = **93.9 GPa** and E_s (surrounding grain boundaries) = **113.21 GPa.**

References:

1. Cappella, B. & Dietler, G. Force-distance curves by atomic force microscopy. *Surf. Sci. Rep.* **34**, 1–104 (1999).

2. Hoffman, D. Measuring the elastic modulus of polymers using the atomic force microscope. *Master's Thesis, Michigan Technol. Univ.* (2010).

3. Tang, B., Ngan, A. H. W. & Pethica, J. B. A method to quantitatively measure the elastic modulus of materials in nanometer scale using atomic force microscopy. *Nanotechnology* **19**, 7 (2008).

4. ASM handbook Volume 2 - Properties and selection: Nonferrous alloys and special-purpose materials. *ASM Met. Handb.* **2**, 1300 (1990).

5. Callister, W. D. Materials science and engineering: An introduction (2nd edition). *Mater. Des.* **12**, 59 (1991).

6. https://www.accuratus.com/silinit.html

7. Pabst, W. & Gregorová, E. V. A. ELASTIC PROPERTIES OF SILICA POLYMORPHS – A REVIEW. *Ceramics-Silikáty* **57**, 167–184 (2013).
